# SCD4 deficiency decreases cardiac steatosis and prevents cardiac remodeling in mice fed a high-fat diet

**DOI:** 10.1016/j.jlr.2024.100612

**Published:** 2024-07-31

**Authors:** Marcin Wolosiewicz, Volodymyr V. Balatskyi, Monika K. Duda, Anna Filip, James M. Ntambi, Viktor O. Navrulin, Pawel Dobrzyn

**Affiliations:** 1 Laboratory of Molecular Medical Biochemistry, Nencki Institute of Experimental Biology, Polish Academy of Sciences, Warsaw, Poland; 2 Department of Clinical Physiology, Centre of Postgraduate Medical Education, Warsaw, Poland; 3 Departments of Biochemistry and Nutritional Sciences, University of Wisconsin-Madison, Madison, WI, USA

**Keywords:** heart, ATGL, lipid droplets, mitochondria, metabolism

## Abstract

Stearoyl-CoA desaturase (SCD) is a lipogenic enzyme that catalyzes formation of the first double bond in the carbon chain of saturated fatty acids. Four isoforms of SCD have been identified in mice, the most poorly characterized of which is SCD4, which is cardiac-specific. In the present study, we investigated the role of SCD4 in systemic and cardiac metabolism. We used WT and global SCD4 KO mice that were fed standard laboratory chow or a high-fat diet (HFD). SCD4 deficiency reduced body adiposity and decreased hyperinsulinemia and hypercholesterolemia in HFD-fed mice. The loss of SCD4 preserved heart morphology in the HFD condition. Lipid accumulation decreased in the myocardium in SCD4-deficient mice and in HL-1 cardiomyocytes with knocked out *Scd4* expression. This was associated with an increase in the rate of lipolysis and, more specifically, adipose triglyceride lipase (ATGL) activity. Possible mechanisms of ATGL activation by SCD4 deficiency include lower protein levels of the ATGL inhibitor G0/G1 switch protein 2 and greater activation by protein kinase A under lipid overload conditions. Moreover, we observed higher intracellular Ca^2+^ levels in HL-1 cells with silenced *Scd4* expression. This may explain the activation of protein kinase A in response to higher Ca^2+^ levels. Additionally, the loss of SCD4 inhibited mitochondrial enlargement, NADH overactivation, and reactive oxygen species overproduction in the heart in HFD-fed mice. In conclusion, SCD4 deficiency activated lipolysis, resulting in a reduction of cardiac steatosis, prevented the induction of left ventricular hypertrophy, and reduced reactive oxygen species levels in the heart in HFD-fed mice.

Lipid metabolism is fundamental to the heart. Fatty acids (FAs) are the primary energy source for cardiomyocytes (1). Stearoyl-CoA desaturase (SCD) is a rate-limiting enzyme for de novo lipid synthesis that catalyzes the formation of a *cis*-double bond at the Δ9 position in the carbon chain of saturated FAs, mainly stearate and palmitate, to synthesize monounsaturated FAs (ie, oleate and palmitoleate, respectively). In mice, four SCD isoforms have been identified: SCD1, SCD2, SCD3, and SCD4 (2, 3, 4, 5). In humans, only two isoforms have been identified: SCD1 and SCD5 (6, 7). Both mouse and human SCD1 are ubiquitously expressed, particularly in adipose tissue and the liver (4, 5, 6). Interestingly, SCD1 is also highly expressed in the human heart (7), although the heart has a limited capacity for lipogenesis and de novo lipid synthesis. SCD2 is also a widespread isoform, except in the liver in adult mice (3, 8). SCD3 is present in the skin, preputial gland, and Harderian gland (4), whereas SCD4 is a heart-specific isoform (5). Human SCD5 is abundantly expressed in the brain and pancreas (7) but not in the heart (9).

*Scd4* expression significantly increased in mice fed a high-carbohydrate fat-free diet, in obese leptin-deficient mice, and in the state of SCD1 deficiency (5, 10). The expression of cardiac *Scd4* is also induced by the liver X receptor agonists, but, opposite to SCD1, it is not repressed by polyunsaturated FAs (5). *Scd1* is a major target gene of leptin in the liver. Leptin represses the expression of *Scd4* but not *Scd1* or *Scd2* in the heart (5). Recently Gan *et al.* (11) showed that the deletion of *Scd4* in the mouse heart reduced adenosine monophosphate-activated protein kinase activation that was induced by myocardial infarction, but the effect on triglyceride (TG) accumulation remains unknown. Additionally, SCD4 deficiency in the post-myocardial infarction myocardium has been shown to reduce reactive oxygen species (ROS) formation via a decrease in protein levels of the nicotinamide adenine dinucleotide phosphate oxidase subunits p47-phox and p91-phox. The loss of SCD4 also reduces cardiac and plasma levels of pro-angiogenic factors, suggesting that SCD4 positively regulates the formation of vascularization in the post-myocardial infarction mouse myocardium (11). The activity of SCD1 and SCD4 has been proposed to overlap in the activation of processes that seek to restore proper cardiac function after myocardial infarction (11).

The role of SCD4 in the regulation of metabolism is poorly understood, but many reports describe multiple beneficial effects of SCD1 downregulation. The loss of SCD1 reduces body adiposity through greater energy expenditure and oxygen consumption and a higher β-oxidation rate in the liver, skeletal muscle, and adipose tissue (12, 13, 14). Insulin signaling increased in the skeletal muscle, the heart, and the brown adipose tissue in SCD1^−/−^ mice (15, 16, 17). In the heart, the loss of SCD1 decreased peroxisome proliferator-activated receptor α (PPARα) activity and polyunsaturated FA content, resulting in a lower rate of FA oxidation (17). SCD1 deficiency promoted cardiac glucose utilization and improved cardiac insulin sensitivity (17, 18). Furthermore, SCD1 deficiency, independent of PPARα, reduced levels of proteins that are involved in FA uptake and lipogenesis but increased the rate of lipolysis, resulting in a reduction of cardiac steatosis in the SCD1^−/−^ mouse heart (19). In obese leptin-deficient mice, *Scd1* KO improved systolic and diastolic heart function by reducing cardiomyocyte apoptosis and cardiac steatosis (10). Conversely, in a model of hyperthyroidism, SCD1 inhibition activated lipogenesis and led to higher TG accumulation (20). *Scd1* KO upregulated the activity of mitochondrial complexes I, II, and III in adipocytes and potentiated beige fat formation (21). Conversely, SCD1 upregulation in cardiomyocytes suppressed saturated FA-induced mitochondrial ROS generation and apoptosis (18). High-fat diet (HFD) feeding decreased mitochondrial adenosine triphosphate production and ΔΨ threshold, which triggered ROS generation in the liver and cardiac mitochondria (22). These factors contribute to cardiac dysfunction.

In the present study, we investigated the role of SCD4 in systemic metabolism, heart function and structure, and cardiac lipid metabolism. Using HFD-fed mice as a model of obesity, we showed that SCD4 deficiency decreased body weight, fat storage, and fasting plasma cholesterol levels. SCD4 deficiency also had a beneficial effect on glucose metabolism, indicated by the suppression of HFD-induced increases in fasting plasma glucose and insulin levels. Furthermore, obese SCD4^−/−^ mice exhibited reductions of cardiac steatosis and ROS levels and were protected against HFD-induced left ventricle remodeling. Lower lipid levels in cardiomyocytes in SCD4^−/−^ mice were accompanied by an increase in lipolysis. These findings indicate that SCD4 is involved in the regulation of cardiac energy metabolism and myocardial function.

## Materials and Methods

### Antibodies

Abhydrolase domain containing 5 (ABHD5; catalog no. sc-100468, working dilution: 1:1000), G0/G1 switch protein 2 (G0S2; catalog no. sc-133423, working dilution: 1:500), and protein kinase A (PKA; catalog no. sc-390548, working dilution: 1:1000) antibodies were obtained from Santa Cruz Biotechnology (Santa Cruz, CA). β-actin (catalog no. A3852, working dilution: 1:50,000) and glyceraldehyde 3-phosphate dehydrogenase (GAPDH; catalog no. MAB374, working dilution: 1:50,000) antibodies were obtained from Merck (Darmstadt, Germany). Adipose triglyceride lipase (ATGL; catalog no. 2138, working dilution: 1:1000) antibody was obtained from Cell Signaling Technology (Hertfordshire, UK). Oxidative phosphorylation (OXPHOS) complexes (catalog no. ab110413, working dilution: 1:2000) antibody was obtained from Abcam (Cambridge, UK). The other chemicals were purchased from Merck, unless otherwise specified.

### Animals

SCD4^−/−^ mice were generated on the C57BL/6 background as previously described (11). Male WT and SCD4^−/−^ mice, both lines bred in the animal facility of the Nencki Institute, at 15 weeks of age were assigned to a group that was fed a standard laboratory diet (chow, Ssniff, catalog no. V1124) or HFD (60% of energy from fat, Ssniff, catalog no. E15742) for 8 weeks to induce obesity. The animals were housed in separate cages by genotype (4–5 mice per cage) in a pathogen-free facility at room temperature under a 12 h/12 h light/dark cycle with ad libitum access to water and food. Body weight and food intake were monitored weekly and every other day, respectively. The mice were euthanized by cervical dislocation at 23 weeks of age. The experiment was repeated twice, with a total of 19–21 mice/group used in the study. All protocols were approved by the First Local Ethical Committee for Animal Experiments in Warsaw.

### Glucose tolerance test

A glucose tolerance test was performed 3 days before the end of the experiment. The animals were fasted for 16 h. Fasting glucose levels were assessed using a drop of blood from the tail end. Glucose levels were measured using glucose strips with an Optium Xido glucose meter. Glucose was then administered intraperitoneally at a dose of 2 g/kg of body weight. Glucose levels were measured after 15, 30, 60, 90, and 120 min.

### Echocardiography

During the last week of the experiment, transthoracic echocardiography was performed using a Vevo 2100 ultrasonograph (VisualSonics, Toronto, Canada) with MS550S linear array transducer (32–56 MHz). During the examination, the mice were anesthetized with continuously administered vaporized 1.5% isoflurane. Short-axis echocardiogram of the left ventricle at the level of the mid-papillary muscle was recorded and used to measure anterior wall thickness in diastole, posterior wall thickness in diastole, and end–diastole diameter (EDD). The data obtained were used to calculate relative wall thickness, ejection fraction (EF), cardiac output and heart rate.

### Blood and tissue sampling

Mice were fasted for 16 h and sacrificed by cervical dislocation. Blood was collected aseptically by direct cardiac puncture and centrifuged at 1,700 *g* at 4°C for 10 min to collect plasma. Samples were aliquoted and stored at −80°C for further analysis. The heart was excised and weighed. The left ventricle was frozen in liquid nitrogen and stored at −80°C. Visceral white adipose tissue was excised and weighed to determine body adiposity.

### Plasma parameters

Plasma glucose, TG, and cholesterol levels were measured using commercial kits (BioSystems, Barcelona, Spain). Free FA levels were determined using the NEFA-HR(2) kit (Wako, Richmond, VA). Plasma insulin levels were assessed using the Rat/Mouse Insulin ELISA kit (Merck).

### Homeostasis model assessment of insulin resistance

The homeostasis model assessment of insulin resistance (HOMA-IR) index was calculated based on fasting plasma glucose level (mmol/L) and insulin level (μU/ml) using the following equation: HOMA-IR = (glucose × insulin)/22.5.

### Isolation and culture of neonatal mouse cardiomyocytes

Newborn (P0-P2) WT and SCD4−/− mice were euthanized by decapitation, hearts were excised and transferred to ice-cold PBS with 20 mM butanedione monoxime, 1000 U/ml penicillin and 1000 μg/ml streptomycin for 30 min. The hearts were then cut and digested overnight at 4°C with 0.25% trypsin, 0.02% ethylenediaminetetraacetic acid, and 20 mM butanedione monoxime. The next day, digestion was continued with 0.75 mg/ml collagenase and 20 mM butanedione monoxime at 37°C for 5 min. This step was repeated twice. After 1 h of preplating on uncoated plates, cardiomyocytes were plated on gelatin-coated plates at a density of 120,000/cm^2^ in 15% fetal bovine serum (FBS), DMEM/M199 (3:1) media. The next day, the medium was replaced with 4% FBS, DMEM/M199 (3:1). Primary cardiomyocytes treatment with 100 μM 18:0 conjugated with FAs free bovine serum albumin (BSA) or BSA (control) for 24 h was performed in 1% FBS, DMEM/M199 (3:1).

### Cardiomyocyte contractility analysis

Primary cardiomyocytes were plated on gelatin-coated CardioECR 48 E-Plate (Agilent Technologies, Santa Clara, CA) at a density of 35,000 cells per well and treated with 18:0 or BSA as described above. Contractility of spontaneously beating cells was recorded using xCELLigence RTCA Cardio system (Agilent Technologies). Data were analyzed using CardioECR Data Analysis software (Agilent Technologies). Beating rate, amplitude, rising time 10%–90% and falling time 90%–10% were measured.

### Culture of HL-1 cells

The heart-derived HL-1 cell line was obtained from W.C. Claycomb (Louisiana State University, New Orleans, LA). Cells were cultured on a gelatin (0.02% [w/v])/fibronectin (10 μg/ml) matrix and maintained in Claycomb medium supplemented with 10% (v/v) FBS, 2 mM glutamine, 0.1 mM norepinephrine, 100 U/ml penicillin, and 100 U/ml streptomycin in a 5% CO_2_ atmosphere at 37°C (23). The medium was changed every 24 h. To knockdown the *Scd4* gene, a 30 nM pool of four target-specific silencing RNA sequences was used (GE HealthCare Dharmacon, Lafayette, CO). JetPRIME transfection reagent (Polyplus, Illkirch, France) was used to deliver silencing RNA (siRNA) to the cells according to the manufacturer’s instructions. Cells were incubated 24 h with transfection medium. Treatment of cells with 100 μM 18:0 or BSA as a control was performed 48 h after transfection.

### Gene expression analysis

Total mRNA from the left ventricle was isolated using a commercial kit (A&A Biotechnology, Gdynia, Poland), treated with DNase (A&A Biotechnology), and reverse transcribed using the RevertAid H Minus First Stand cDNA Synthesis Kit (Thermo Fisher Scientific, Pittsburgh, PA) according to the manufacturer’s instructions. Quantitative real-time PCR was performed using the CFX Connect Real-Time PCR Detection System (Bio-Rad, Hercules, CA) with SsoAdvanced Universal SYBR Green Supermix (Bio-Rad, Hercules, CA). The relative expression of each gene was determined using the 2^-ΔΔCt^ method, with 60S ribosomal protein L32 (*Rpl32*) as the reference gene. The list of primers is shown in Supplemental Table S1.

### Transmission electron microscopy

A portion of the left ventricular free wall tissue was fixed in 1% osmium tetroxide, dehydrated, and stained with 1% uranyl acetate in 70% ethanol. The sample was then incubated in a mixture of ethanol/propylene (1/1 [v/v]), followed by pure propylene oxide. The tissue was embedded in Epon resin. Ultra-thin sections were collected on grids and post stained with uranyl acetate and Reynold’s lead citrate. Electron micrographs were obtained with a Morada camera and JEM 1400 transmission electron microscope at 80 kV (JEOL, Tokyo, Japan) in the Laboratory of Electron Microscopy, Nencki Institute of Experimental Biology, Warsaw, Poland. The number and area of mitochondria, length of sarcomeres, and area and number of lipid droplets (LDs) were calculated from images at 6,000× magnification using ImageJ software (National Institutes of Health, Bethesda, MD). The relative frequency of LDs was calculated as the percentage of the number of LDs included in each bin covering 0.05 μm^2^ relative to the total number of LDs in all bins.

### Histology

For paraffin-embedded sections, the heart was fixed in 7% formaldehyde, followed by 10% formaldehyde and dehydration. The tissue was incubated in xylene and then in paraffin for embedding. Using a Zeiss Hyrax M55 microtome (Carl Zeiss, Oberkochen, Germany), heart sections (6 μm thick) were collected. For cryosectioning, the heart was cryopreserved in 30% sucrose and embedded in optimum cutting temperature medium. The 10-μm-thick sections were collected with a Microm HM550 cryostat (Thermo Fisher Scientific). The analysis was conducted in the Laboratory of Electron Microscopy, Nencki Institute of Experimental Biology, Warsaw, Poland.

### Hematoxylin/eosin staining

Paraffin sections of the heart were deparaffinized and rehydrated. Mayer’s hematoxylin solution was used to stain nuclei. An aqueous solution of eosin Y was then used to counterstain the cytoplasm. Tissue sections were dehydrated, mounted, and observed with a 60× magnification objective using an Olympus BX41 microscope (Olympus, Tokyo, Japan).

### Reactive oxygen species staining

Reactive oxygen species staining was performed as described previously (24) using dihydroethidium (Cayman, Ann Arbor, MI). Briefly, frozen heart sections were rinsed with ddH_2_O and immediately placed in 5 μM dihydroethidium while protected from light. After incubation, the dye was washed in ddH_2_O. Images were captured with a 10× magnification objective using a Texas Red filter, and fluorescence intensity was analyzed using ImageJ software.

### NADH dehydrogenase activity

The NADH dehydrogenase complex (OXPHOS I) was stained as described elsewhere (25). Briefly, frozen sections were washed in PBS and stained with 0.625 mg/ml NADH and 1.5 mM nitro blue tetrazolium in 0.1 M PBS, pH 7.0. The sections were then washed in PBS, dehydrated, and mounted. The stained sections were observed with a 20× magnification objective, and the mean intensity (*I*_mean_) and maximal intensity (*I*_max_) were measured with ImageJ software. OXPHOS I activity was derived from log_10_(*I*_max_/*I*_mean_).

### Neutral lipid staining

Neutral lipids were stained using Oil Red O (ORO) dye. Stock solution of the dye was prepared by dissolving 0.4 g ORO in 100 ml of 99% isopropanol overnight and filtered using a 0.2 μm filter. A working solution of ORO was obtained by mixing the stock solution with ddH_2_O in a 3:2 (v/v) proportion, followed by 0.2 μm filtration. HL-1 cells were fixed in 4% formaldehyde in PBS before staining. HL-1 cells were then incubated in 60% isopropanol, followed by staining in working solution, and the unabsorbed dye was washed five times in ddH_2_O. To assess lipid accumulation in HL-1 cells, the dye was extracted using 99% isopropanol, and absorbance was measured at 518 nm using an Infinite M200 Pro plate reader (Tecan, Männedorf, Switzerland).

### BODIPY staining

Primary cardiomyocytes were fixed with 4% paraformaldehyde and permeabilized with 0.2% Triton X-100. Cell staining was performed with 1 μg/ml BODIPY (Thermo Fisher Scientific), and nuclei were counterstained with DAPI. Images were captured with Olympus BX41 microscope equipped with 60× objective. Quantification of LDs was performed using ImageJ software.

### Western blot

The left ventricle was homogenized in ice-cold lysis buffer that contained 20 mM Tris-HCl (pH 7.4), 2 mM ethylene glycol-bis(2-aminoethylether)-*N*,N,*N′*,*N′*-tetraacetic acid, 2 mM ethylenediaminetetraacetic acid, 2 mM Na_3_VO_4_, 1 mM phenylmethylsulfonyl fluoride, 10 mM β-mercaptoethanol, 10 μg/ml leupeptin, 5 μg/ml pepstatin A, and 2 μg/ml aprotinin and centrifuged at 10,000 *g* for 15 min at 4°C. The protein content in the lysates was determined using the Bio-Rad Protein Assay with BSA as a reference. Protein samples were separated using 10% sodium dodecyl sulfate-PAGE gels and transferred to polyvinylidene difluoride membranes (Millipore, Billerica, MA). Western blot analysis was performed using appropriate antibodies. The activity of horseradish peroxidase-conjugated secondary antibodies was detected using SuperSignal West Pico PLUS Chemiluminescent Substrate (Thermo Fisher Scientific) and quantified by densitometry. Protein levels are expressed relative to the abundance of GAPDH.

### Lipid analysis

Lipids were extracted from HL-1 cells, and the left ventricles according to the method of Bligh and Dyer (26). The extracted lipids were separated by thin-layer chromatography on silica gel 60 plates (Merck) in heptane/isopropyl ether/glacial acetic acid (60/40/3 [v/v/v]) with authentic standards. To visualize lipid bands, the plate was soaked in a water mixture that contained 10% cupric sulfate and 8% phosphoric acid and then burned in at 140°C for 20 min. The lipids were then quantified by densitometry.

For the quantification of TGs, band that corresponded to TGs was scraped off the plate and transferred to screw-cap glass tubes that contained methylnonadecanoic acid as an internal standard. The FAs were then transmethylated in the presence of 14% boron trifluoride in methanol. The resulting methyl esters were extracted with hexane and analyzed by gas chromatography-mass spectrometry using the Agilent 7890A-5975CGC-MS system/Agilent 19091N-205 capillary column (Agilent Technologies). The total content of TGs was calculated from the content of individual FAs.

### Lipolysis assay

ATGL activity was measured as described previously (27) with modifications. Briefly, left ventricular samples were homogenized using TissueLyser II (Qiagen, Germantown, MD) in Buffer A (50 mM Hepes [pH 7.2], 100 mM NaCl, 0.5 mM DTT, 2% dimethylsulfoxide, and 0.1% Triton X-100) and then centrifugated at 500 *g* for 5 min at 4°C. HL-1 cells and primary cardiomyocytes were resuspended in Buffer A and passed 10 times through Hamilton syringe, then snap frozen in liquid nitrogen and thawed three times at 37°C. The supernatant was collected as a source of ATGL enzyme, and protein concentration was measured. ATGL activity was determined using 1 μM EnzChek lipase substrate (Thermo Fisher Scientific). Fluorescence (excitation: 485 nm, emission: 510 nm) was recorded every 60 s using a Tecan Infinite M200 Pro plate reader. Enzyme activity was calculated using the linear part of the velocity curve.

### Cytoplasmatic calcium measurement

Intracellular calcium ion concentration was measured using the Fluo-4 Direct Calcium Assay Kit (Thermo Fisher Scientific) according to the manufacturer’s instructions. Briefly, 48 h after transfection, HL-1 cells were incubated with Fluo-4 Direct loading solution for 60 min. Fluorescence intensity was measured using a Tecan Infinite M200 Pro plate reader (excitation: 494 nm, emission: 516 nm). Cell nuclei were then stained with Hoechst dye for 5 min and measured using a Tecan Infinite M200 Pro plate reader (excitation: 361 nm, emission: 497 nm). The ratio of Fluo-4 Direct to Hoechst fluorescence intensity was calculated as normalized Fluo-4 Direct fluorescence.

### Statistical analysis

The data are expressed as the mean ± SD, with n = 10–12 mice/group. The data were analyzed using two-way analysis of variance (ANOVA), followed by the Holm-Sidak multiple-comparison *post hoc* test. For HL-1 cells, the results from three independent experiments are presented, and a two-tailed *t* test was performed. Values of *P* < 0.05 were considered statistically significant. The statistical analysis was performed using Prism 8.3.0 software (GraphPad, San Diego, CA; https://www.graphpad.com/).

## Results

### Lack of SCD4 protects against HFD-induced obesity

**Fig. 1:**
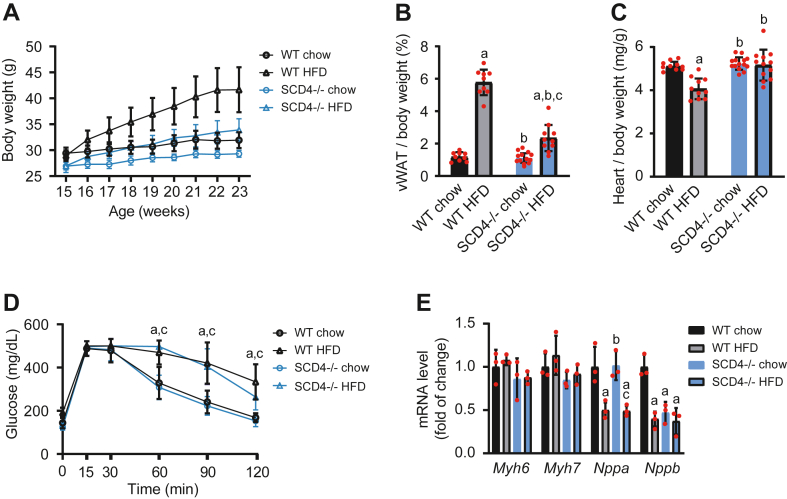
A: Effect of SCD4 deficiency on body weight in mice. WT and SCD4^−/−^ mice were fed chow or a high-fat diet (HFD). During the 8-week experiment, body weight was monitored weekly. B: Adiposity in mice, calculated as the ratio of visceral white adipose tissue (vWAT) weight to body weight, expressed as a percentage. C: Ratio of heart weight to body weight. D: Glucose tolerance in mice was analyzed at the end of the experiment using an intraperitoneal glucose tolerance test. E: Effect of SCD4 deficiency on the expression of genes that are related to heart dysfunction. mRNA levels of α and β myosin heavy chain (*Myh6* and *Myh7*), atrium natriuretic peptide (*Nppa*), and B-type natriuretic peptide (*Nppb*) were analyzed using quantitative real-time PCR and the 2^-ΔΔCt^ method. The data are expressed as the mean ± SD. n = 10–12 mice/group. ^a^*p* < 0.05, *versus* WT chow; ^b^*p* < 0.05, *versus* WT HFD; ^c^*p* < 0.05, *versus* SCD4^−/−^ chow.

SCD1-deficient mice are protected against HFD-induced obesity (12). To test whether SCD4 deficiency has a similar effect, we fed WT mice and SCD4^−/−^ mice a standard chow diet or HFD to induce obesity. Initially, SCD4^−/−^ mice had a significantly lower basal body weight compared with WT mice (26.9 ± 1.0 g and 29.2 ± 1.1 g, respectively). Moreover, HFD-fed SCD4^−/−^ mice had lower weight gain over the 8 weeks of the HFD compared with WT mice (26% and 44%, respectively; Fig. 1A). These changes did not depend on food intake, which was similar for WT and SCD4^−/−^ mice (Supplemental Fig. S1). SCD4^−/−^ mice that were fed the HFD accumulated 68% less visceral white adipose tissue compared with WT mice (0.8 ± 0.3 g and 2.5 ± 0.5 g, respectively), and thus had a lower body adiposity index (Fig. 1B). The ratio of heart weight to body weight decreased in HFD-fed WT mice but was unchanged in HFD-fed SCD4^−/−^ mice (Fig. 1C). The glucose tolerance test showed that both genotypes developed glucose intolerance during HFD feeding (Fig. 1D). However, SCD4 deficiency abolished the HFD-induced increase in fasting plasma glucose levels and lowered insulin levels after HFD feeding compared with HFD-fed WT mice, resulting in an improved HOMA-IR index (Table 1). Moreover, SCD4^−/−^ mice had lower plasma cholesterol levels on both the chow diet and HFD. No changes were observed in plasma TG and nonesterified FA concentrations (Table 1). In conclusion, SCD4^−/−^ mice had lower basal body weight, lower weight gain, and lower body adiposity. Moreover, SCD4 deficiency preserved fasting glucose levels and reduced hyperinsulinemia and hypercholesterolemia.

**Table 1:** Plasma parameters

Parameter	WT Chow	WT HFD	SCD4^−/−^ Chow	SCD4^−/−^ HFD
Glucose [mg/dl]	174.0 ± 28.1	222.2 ± 25.4^a^	168.5 ± 31.8^b^	190.7 ± 23.8
Insulin [ng/ml]	1.5 ± 0.1	4.6 ± 0.2^a^	1.4 ± 0.1^b^	2.2 ± 0.5^a^^,^^b^^,^^c^
HOMA-IR index	17.9 ± 2.5	72.1 ± 10.9^a^	17.0 ± 1.3^b^	29.3 ± 6.5^a^^,^^b^^,^^c^
Cholesterol [mg/dl]	85.5 ± 8.3	149.1 ± 17.6^a^	71.5 ± 11.6^a^^,^^b^	123.8 ± 21.8^a^^,^^b^^,^^c^
Triglyceride [mg/dl]	130.9 ± 35.9	131.2 ± 28.2	113.5 ± 21.5	130.3 ± 25.4
Nonesterified fatty acids [mg/dl]	24.1 ± 7.3	20.9 ± 5.3	23.0 ± 6.0	22.3 ± 5.0

The data are expressed as mean ± SD, n = 10–12 mice/group.

a *P* < 0.05, versus WT chow.

b *P* < 0.05, versus WT HFD.

c *P* < 0.05, versus SCD4^−/−^ chow.

### SCD4 deficiency affects cardiac morphology in HFD-fed mice

Transthoracic echocardiography was performed to assess left ventricular morphology and function. Heart rate, EF, and CO were similar among groups (Table 2). No differences in parameters of left ventricular morphology were found between chow-fed SCD4^−/−^ mice and WT mice, except for a significant decrease in EDD and end-systole diameter (ESD). Eight-week HFD feeding initiated concentric remodeling in WT mice, indicated by a decrease in EDD and ESD and an increase in anterior wall thickness in diastole (by 9.6%) and posterior wall thickness in diastole (by 13%), leading to a significant increase in relative wall thickness (Table 2). Interestingly, HFD feeding did not induce left ventricular remodeling in SCD4^−/−^ mice compared with chow-fed SCD4^−/−^ mice (Table 2). Next, we measured the expression of genes that are related to cardiac remodeling, including α and β myosin heavy chain (MHC; *Myh6* and *Myh7*), atrium natriuretic peptide (*Nppa*), and B-type natriuretic peptide (*Nppb*). The expression of *Myh6* and *Myh7* was similar among groups. HFD feeding decreased *Nppa* mRNA levels in both WT and SCD4^−/−^ mice. *Nppb* expression decreased in the heart in SCD4^−/−^ mice that were fed a chow diet compared with WT mice. In contrast to WT mice, however, it was unaltered by HFD feeding in SCD4^−/−^ mice (Fig. 1E). SCD4 deficiency did not affect cardiomyocyte morphology or sarcomere length in either chow- and HFD-fed groups (Supplemental Fig. S2). In summary, the lack of SCD4 caused minor changes in left ventricle morphology but prevented its structural changes in the HFD condition.

**Table 2:** Echocardiographic analysis of left ventricular function and structure in WT and SCD4^−/−^ mice

Parameter	WT Chow	WT HFD	SCD4−/− Chow	SCD4^−/−^ HFD
HR [bpm]	361 ± 18	333 ± 39	336 ± 50	345 ± 38
AWTd [mm]	0.83 ± 0.03	0.91 ± 0.06	0.87 ± 0.09	0.91 ± 0.09
PWTd [mm]	0.91 ± 0.05	1.03 ± 0.05	0.95 ± 0.07	1.04 ± 0.12
EDD [mm]	4.10 ± 0.12	3.73 ± 0.17^a^	3.66 ± 0.21^a^	3.67 ± 0.36^a^
ESD [mm]	2.72 ± 0.14	2.53 ± 0.16^a^	2.27 ± 0.33^a^	2.28 ± 0.24^a^
RWT	0.44 ± 0.02	0.55 ± 0.01^a^	0.52 ± 0.04	0.57 ± 0.09^a^
EF [%]	70.67 ± 1.51	68.67 ± 1.37	75.00 ± 11.24	75.67 ± 2.73
CO [mL/min]	17.75 ± 1.23	12.64 ± 2.86	12.91 ± 2.82	13.81 ± 4.20

AWTd, anterior wall thickness in diastole; CO, cardiac output; EDD, end-diastole diameter; EF, ejection fraction; ESD, end-systole diameter; HR, heart rate; PWTd, posterior wall thickness in diastole; RWT, relative wall thickness. The data are expressed as mean ± SD, n = 5–6 mice/group.

a *P* < 0.05, versus WT chow.

**Fig. 2:**
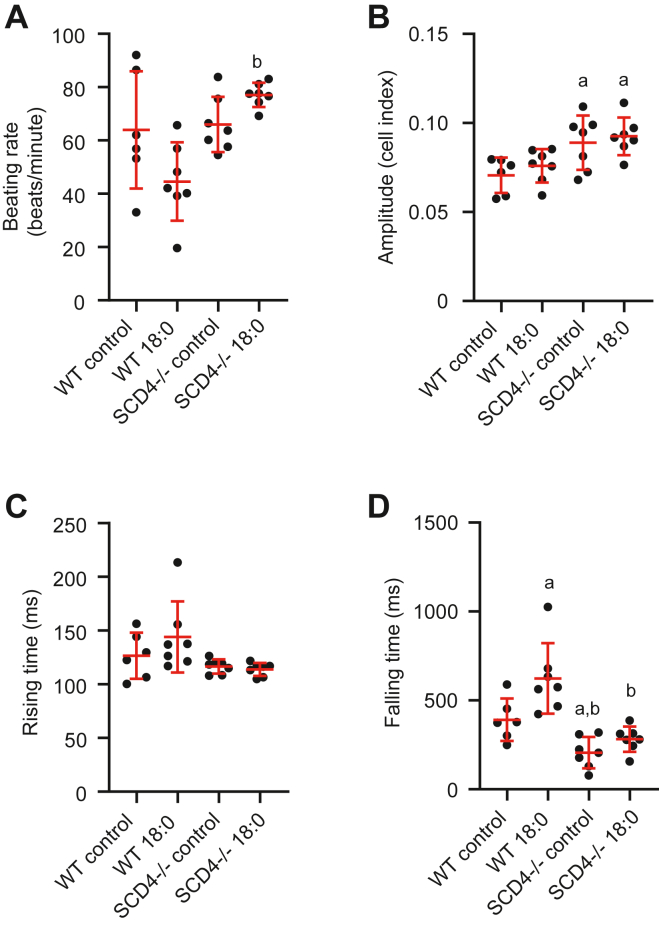
Effect of SCD4 deficiency on contractile properties of neonatal cardiomyocytes. A: Spontaneous beating rate of primary cardiomyocytes treated with stearic acid. B: Amplitude of cardiomyocyte contraction. C: Rising time (time for the cell index to increase from 10% to 90% of amplitude). D: Falling time (time for the cell index to decrease from 90% to 10% of amplitude). ^a^*p* < 0.05, *versus*. WT control; ^b^*p* < 0.05, *versus* WT 18:0. The data are expressed as the mean ± SD. n = 6–7 independent measurements.

Next, we analyzed the contractility of neonatal cardiomyocytes after treatment with stearic acid. In the basal state, loss of SCD4 did not affect beating rate and rising time of cardiomyocytes, but increased amplitude of contraction and decreased falling time (Fig. 2A–D). After 18:0 treatment, the beating rate of SCD4-deficient cardiomyocytes was increased compared to the corresponding WT cells (Fig. 2A). Stearic acid did not affect amplitude of contraction and rising time in either WT or SCD4-deficient cardiomyocytes (Fig. 2B, C). The 18:0 treatment impaired the relaxation of WT cardiomyocytes, as evidenced by a prolonged falling time (Fig. 2A).The falling time was unchanged in SCD4-deficient cardiomyocytes after 18:0 treatment and was decreased compared to WT cells treated with 18:0 (Fig. 2D). In summary, the lack of SCD4 caused minor changes in left ventricle morphology but prevented its structural changes in the HFD condition and preserved contractile function of cardiomyocytes exposed to 18:0.

### Cardiac lipid accumulation decreased in SCD4-deficient mice

**Fig. 3:**
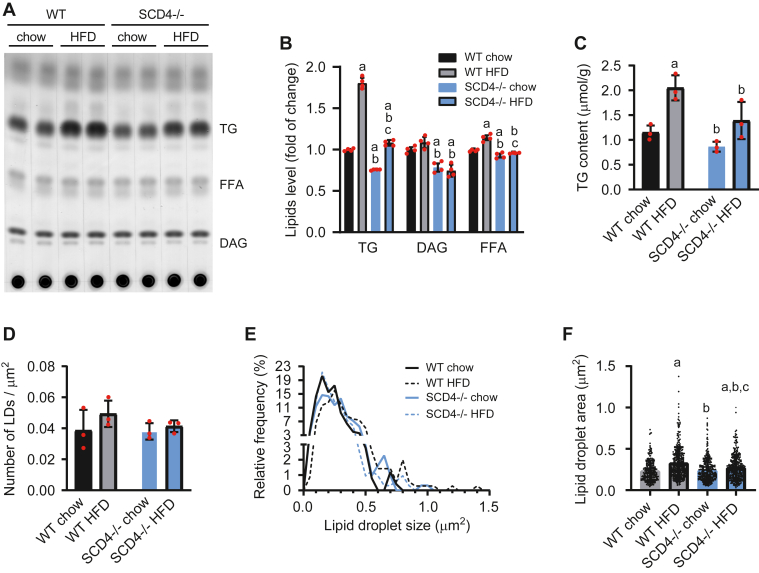
Effect of SCD4 deficiency on myocardial lipid accumulation. A: Representative thin-layer chromatography plate of separated neutral lipids. Lipids were isolated according to the method of Bligh and Dyer (1959). B: After soaking in a mixture of 10% cupric sulfate and 8% phosphoric acid, the plate was burned in at 140°C, and lipids were quantified by densitometry. C: Triglyceride (TG) content was analyzed using gas chromatography-mass spectrometry. D: The number of lipid droplets (LDs) and E: LD size distribution were determined using transmission electron microscopy images at 6,000× magnification. F: Mean lipid droplet size measured on TEM images at 6,000× magnification (n > 250). The data are expressed as the mean ± SD. n = 10–12 mice/group. ^a^*p* < 0.05, *versus* WT chow; ^b^*p* < 0.05, *versus*. WT HFD; ^c^*p* < 0.05, *versus*. SCD4^−/−^ chow. HFD, high-fat diet.

**Fig. 4:**
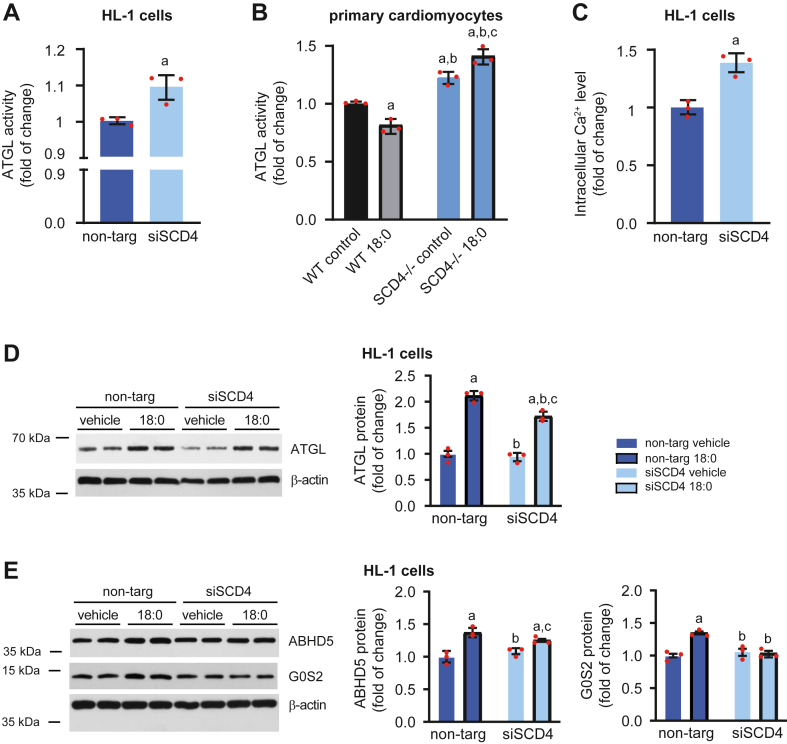
Impact of *Scd4* downregulation on lipolysis in HL-1 cells and primary cardiomyocytes. A: ATGL activity in HL-1 cells with silenced *Scd4* expression. B: ATGL activity in primary SCD4-deficient cardiomyocytes treated with stearic acid (C18:0). C: Intracellular calcium concentration in HL-1 cells was measured using the Fluo-4 Direct Calcium Assay Kit according to the manufacturer’s instructions. D: ATGL, E: ABHD5 and G0S2 protein levels in HL-1 cells and results of densitometric analysis. The data are expressed as the mean ± SD. n = 3 independent experiments. (A, C, D, E) ^a^*p* < 0.05, *versus* non-targ vehicle; ^b^*p* < 0.05, *versus* non-targ 18:0; ^c^*p* < 0.05, *versus* siSCD4 vehicle. (B) ^a^*p* < 0.05, *versus* WT control; ^b^*p* < 0.05, *versus* WT 18:0; ^c^*p* < 0.05, *versus* SCD4^−/−^ control. ATGL, adipose triglyceride lipase.

To examine how the loss of *Scd4* affects the expression of *Scd1* and *Scd2* in the heart, we performed quantitative real-time PCR. No differences in the expression of *Scd* isoforms were observed between WT and SCD4^−/−^ mice, as well as between chow-fed and HFD-fed mice (Supplemental Fig. S3). Next, to assess the effect of *Scd4* deletion on cardiac lipids, we performed lipid content analysis using thin-layer chromatography. The results showed a protective effect of SCD4 deficiency against the HFD-stimulated cardiac accumulation of TG, the main component of LDs (Fig. 3A, B). This was confirmed by gas chromatography-mass spectrometry, which showed a decrease in TG content (by 32%) in the heart in SCD4^−/−^ mice that were fed the HFD compared with HFD-fed WT mice (Fig. 3C). The analysis of transmission electron microscopy images (Fig. 4A) showed that the number of LDs per tissue area was similar in all groups (Fig. 3D). However, SCD4 deficiency decreased the HFD-induced growth of LDs, indicated by the LD size distribution (Fig. 3E). The mean cardiac LD area was 17% lower in HFD-fed SCD4^−/−^ mice compared with HFD-fed WT mice and was similar in chow-fed SCD4^−/−^ and WT mice (Fig. 3F). Furthermore, SCD4 deficiency decreased diglyceride and FFA content in the heart (Fig. 3A, B).

### Scd4 silencing reduced FA-stimulated lipid accumulation in HL-1 cardiomyocytes

**Fig. 5:**
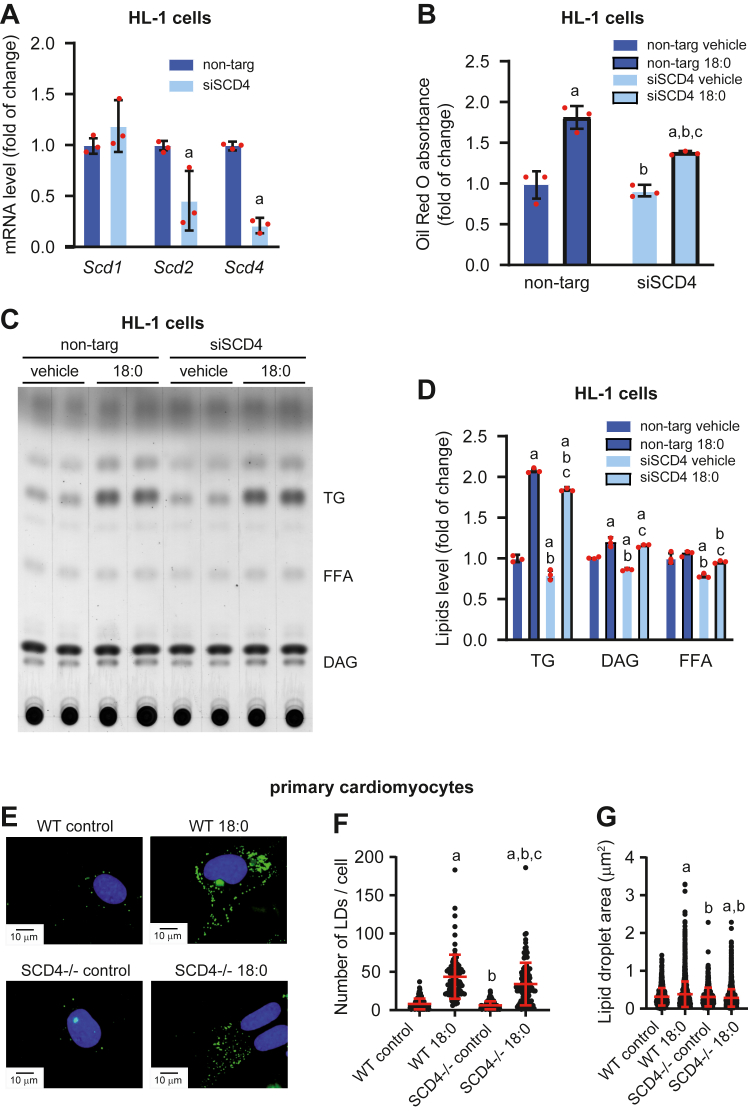
Effect of *Scd4* downregulation in HL-1 cells and primary neonatal cardiomyocytes on lipid accumulation. A: *Scd1*, *Scd2*, and *Scd4* mRNA levels in HL-1 cells after *Scd4* silencing. B: Level of accumulated lipids, based on Oil Red O dye absorption in HL-1 cells that were treated with stearic acid (C18:0). C: Representative thin-layer chromatographic plate of separated neutral lipids from HL-1 cells and D: results of the plate densitometric analysis. E: Representative images of lipid droplets (LDs) staining using BODIPY in primary SCD4-deficient cardiomyocytes. Representative images at 60× magnification are presented. Scale bar = 10 μm. F: The number of LDs and G: LDs size measured in primary neonatal SCD4-defcient cardiomyocytes treated with stearic acid (C18:0). The data are expressed as the mean ± SD. (A–D) n = 3 independent experiments. ^a^*p* < 0.05, *versus* non-targ vehicle; ^b^*p* < 0.05, *versus* non-targ 18:0; ^c^*p* < 0.05, *versus* siSCD4 vehicle. (E-G) n = 3 independent cardiomyocytes isolation. At least 20 cells from each isolation in each experimental condition were analyzed. ^a^*p* < 0.05, *versus* WT control; ^b^*p* < 0.05, *versus* WT 18:0; ^c^*p* < 0.05, *versus* SCD4−/− control.

To confirm that the downregulation of *Scd4* expression affects the reduction of lipid content in cardiomyocytes, we knocked down the *Scd4* gene in HL-1 cardiomyocytes using siRNA (siSCD4) and non-targeting RNA (non-targ) as controls. *Scd4* mRNA levels were 79% lower in siSCD4-treated cells compared with the control (Fig. 5A). Interestingly, *Scd2* expression was also downregulated after *Scd4* gene silencing, whereas *Scd1* was unchanged (Fig. 5A). To stimulate lipid accumulation, we treated HL-1 cells with stearic acid (C18:0). FA treatment induced LD accumulation, indicated by a higher absorbance of ORO dye, whereas silencing the *Scd4* gene decreased the level of accumulated lipids (Fig. 5B), especially TGs (Fig. 5C, D). Decreased number (Fig. 5E, F) and size (Fig. 5G) of LDs were also observed in primary SCD4-deficient cardiomyocytes treated with 18:0. In summary, downregulation of the *Scd4* gene decreased FA-stimulated lipid accumulation in HL-1 cells and neonatal SCD4-deficient cardiomyocytes.

### *Scd4* downregulation increases lipolysis in mouse cardiomyocytes

**Fig. 6:**
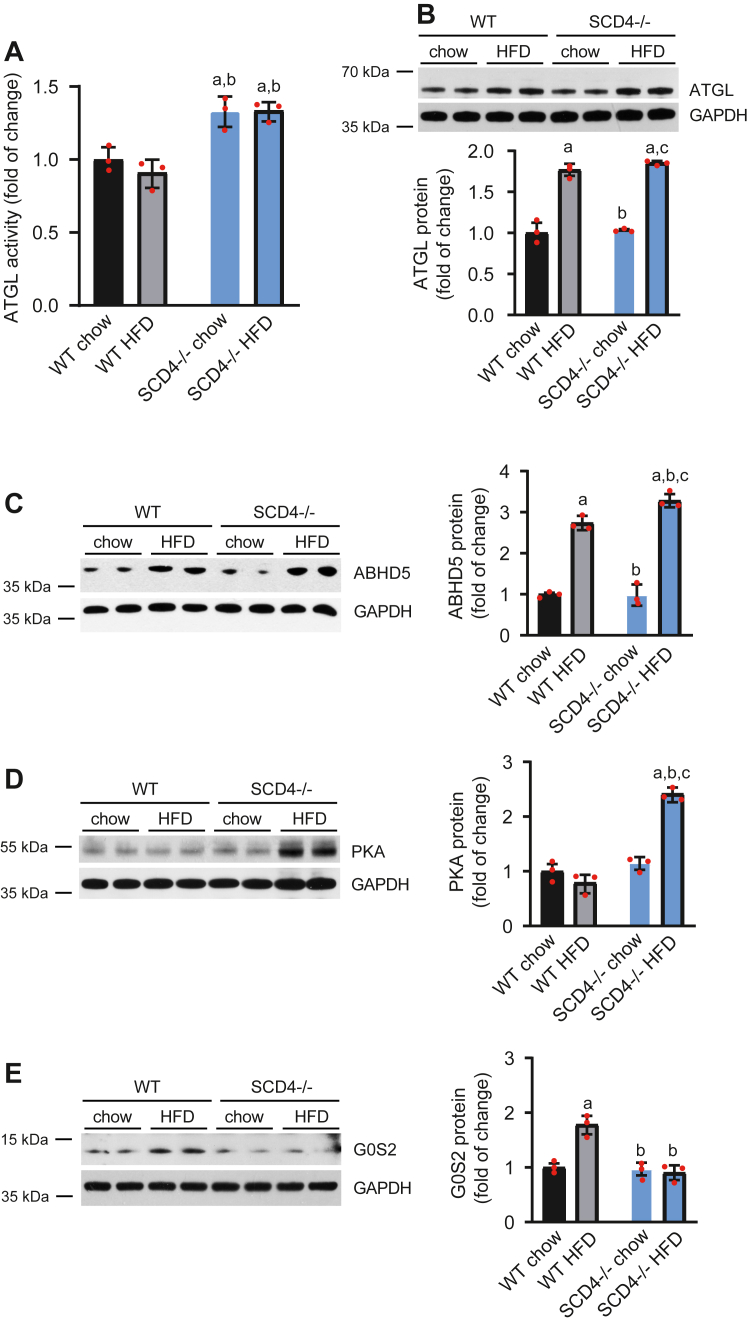
SCD4 deficiency affects lipolysis in the mouse heart. A: Adipose triglyceride lipase (ATGL) activity. Using a triglyceride analog that becomes fluorescent after hydrolysis, we measured ATGL activity as described in the Materials and Methods. B: ATGL, C: abhydrolase domain-containing protein 5 (ABHD5), D: protein kinase A (PKA), and E: G0/G1 switch gene 2 (G0S2) protein content and results of the densitometric analysis. The data are expressed as the mean ± SD. n = 10–12 mice/group. ^a^*p* < 0.05, *versus* WT chow; ^b^*p* < 0.05, *versus* WT HFD; ^c^*p* < 0.05, *versus* SCD4^−/−^ chow. ATGL, adipose triglyceride lipase; HFD, high-fat diet.

To determine how SCD4 deficiency leads to the lower accumulation of LDs in the heart, we measured the activity of ATGL, a rate limiting enzyme of TG hydrolysis. ATGL activity was significantly higher in cardiomyocytes in SCD4^−/−^ mice compared with WT mice (Fig. 6A). Next, we measured levels of proteins that are involved in lipolysis, including ATGL, G0S2, ABHD5, and PKA. HFD feeding increased ATGL protein levels in the heart in WT and SCD4^−/−^ mice (Fig. 6B). However, HFD-fed SCD4^−/−^ mice had higher levels of ABHD5 (Fig. 6C) and PKA (Fig. 6D), which are activators of ATGL, and suppressed the HFD-induced increase in protein levels of G0S2 (Fig. 6E), an inhibitor of ATGL, compared with HFD-fed WT mice. These results showed that the lack of SCD4 increased the rate of lipolysis in the heart in HFD-fed mice.

To test whether silencing the *Scd4* gene also affects the rate of lipolysis in HL-1 cells and primary SCD4-defcient cardiomyocytes, we performed an ATGL activity assay. As in the heart in SCD4−/− mice, *Scd4* silencing significantly increased ATGL activity in HL-1 cells (Fig. 4A) and primary SCD4-deficient cardiomyocytes (Fig. 4B). Increased ATGL activity in HL-1 cells was accompanied by higher intracellular Ca^2+^ levels (Fig. 4C), which has been shown to increase lipolysis (28, 29, 30). C18:0 treatment increased ATGL and ABHD5 protein levels in both control and siSCD4-treated cells (Fig. 4D, E). However, silencing the *Scd4* gene prevented the C18:0-induced upregulation of G0S2 (Fig. 4E). These data confirmed that low *Scd4* expression increased the rate of lipolysis in cardiomyocytes.

### SCD4 deficiency suppresses HFD-induced mitochondrion enlargement

**Fig. 7:**
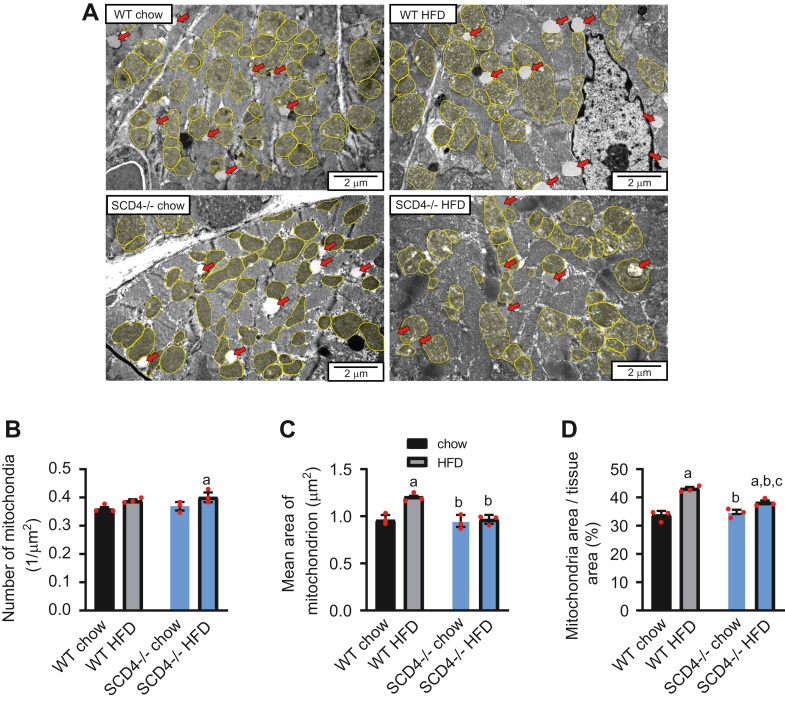
Analysis of cardiac mitochondria by transmission electron microscopy. A: Representative images of mitochondria (yellow) and lipid droplets (red arrows) in the left ventricle. Representative images at 150,00× magnification are presented. Scale bar = 2 μm. B: Mitochondrial density (n > 600), C: average area of a single mitochondrion, and D: percentage of tissue area occupied by mitochondria, presented as a percentage of tissue (n > 250). The data are expressed as the mean ± SD. n = 3 mice/group. ^a^*p* < 0.05, *versus*. WT chow; ^b^*p* < 0.05, *versus* WT HFD; ^c^*p* < 0.05, *versus*. SCD4^−/−^ chow. HFD, high-fat diet.

Mitochondrial dysfunction is a triggering mechanism of HFD-induced cardiac hypertrophy (31, 32). To investigate whether SCD4 deletion affects mitochondria, transmission electron microscopy imaging was performed (Fig. 7A). The number of mitochondria per area of cardiac tissue was similar between WT and SCD4^−/−^ mice that were fed chow or the HFD (Fig. 7B), but SCD4 deficiency repressed the HFD-induced enlargement of mitochondria (presented as the mean area of a single mitochondrion) in WT mice (Fig. 7C). Moreover, SCD4 deficiency significantly attenuated the HFD-induced increase in the percentage of mitochondria in the heart (Fig. 7D).

### SCD4 deficiency reduces NADH dehydrogenase hyperactivity and ROS overproduction

**Fig. 8:**
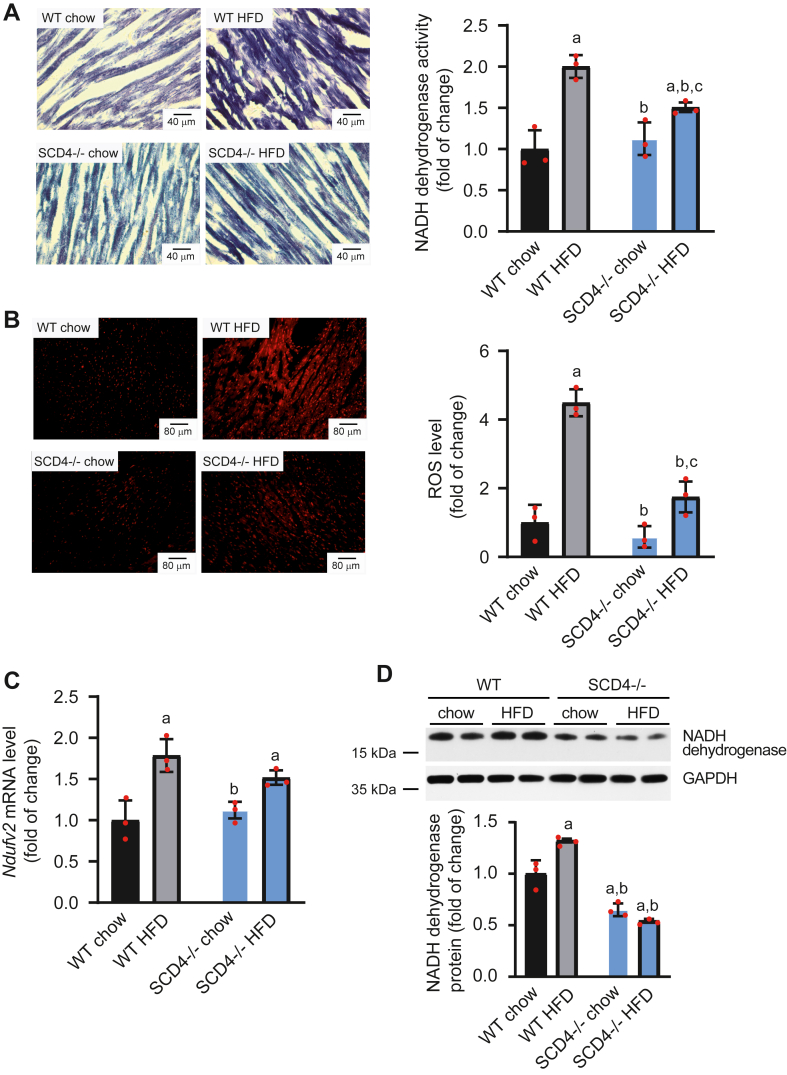
Impact of SCD4 deficiency on mitochondrial NADH dehydrogenase activity and reactive oxygen species (ROS) production. A: NADH dehydrogenase activity and representative images of NADH dehydrogenase activity-dependent staining of the mouse left ventricle. Cryosections (10 μm thick) were stained with NADH and nitro blue tetrazolium. The stained sections were observed with a 20× magnification objective. Scale bar = 40 μm. B: Reactive oxygen species level and representative images of mouse left ventricle staining with ROS-sensitive dye. Images were captured at 10× magnification. Scale bar = 80 μm. C: *Ndufv2* mRNA levels in the heart were analyzed using quantitative real-time PCR and the 2^-ΔΔCt^ method. D: NADH dehydrogenase subunit B8 protein level in the left ventricle of the mouse heart. The data are expressed as the mean ± SD. n = 3 mice/group in A and B. n = 10–12 mice/group in C and D. ^a^*P* < 0.05, *versus* WT chow; ^b^*p* < 0.05, *versus*. WT HFD; ^c^*p* < 0.05, *versus* SCD4^−/−^ chow. HFD, high-fat diet.

To follow the observed changes in mitochondrial morphology, we performed a staining-dependent analysis of the activity of NADH dehydrogenase, the first complex of the electron transport chain. In addition to its physiological role in NADH oxidation, NADH dehydrogenase is responsible for the production of ROS. Therefore, we stained cardiac tissue with a ROS-sensitive dye. Quantitative image analysis showed that SCD4-deficient cardiomyocytes in HFD-fed mice were characterized by lower NADH dehydrogenase activity (by 25%; Fig. 8A) and a decrease in ROS levels (by 61%; Fig. 8B) compared with HFD-fed WT mice. The expression of *Ndufv2*, which encodes NADH dehydrogenase, was unchanged in the heart in HFD-fed SCD4^−/−^ mice compared with chow-fed SCD4^−/−^ mice. In contrast, HFD feeding in WT mice increased expression of the *Ndufv2* gene (Fig. 8C). SCD4 deficiency decreased NADH dehydrogenase protein levels compared with hearts in chow-fed WT mice and prevented the HFD-induced increase in NADH dehydrogenase protein levels (Fig. 8D). Altogether, SCD4 deficiency suppressed HFD-induced upregulation of the *Ndufv2* gene and NADH dehydrogenase protein levels and attenuated NADH dehydrogenase hyperactivity and ROS overproduction in cardiomyocytes.

## Discussion

SCD4 was found to be a heart-specific isoform (5), but our study showed that its global KO also has systemic effects. Like other SCD-deficient mouse models (12, 33), HFD-fed SCD4^−/−^ mice had lower basal body weight, lower weight gain, lower fat accumulation, and lower obesity. Like SCD2^−/−^ mice (33), SCD4^−/−^ mice had similar food intake in the chow-fed and HFD-fed groups. However, SCD1^−/−^ mice had higher food intake, accompanied by increases in energy expenditure and oxygen consumption to maintain body temperature, likely because of the lack of fur and impairment in sebaceous lipids (34, 35). SCD4^−/−^ mice had normal fur, so the reasons for the phenotypic changes that were observed in this mouse model are currently unknown and require further study. One possibility is that *Scd4* is also expressed in other tissues that have not yet been studied. For example, some studies showed that the inhibition of intestinal fat absorption can reduce body obesity adiposity. Intestinal SCD1 has been shown to modulate lipid content and composition not only in intestinal tissues but also in plasma and the liver, possibly through the production of new signaling lipids (36). Thus, if *Scd4* is expressed in the intestine, then its deletion may have a similar systemic effect.

The heart has also been suggested to be an endocrine organ. In addition to atrial natriuretic factor and brain natriuretic peptide, other polypeptide hormones are expressed in the heart that likely act on the myocardium in a paracrine or autocrine fashion. These include the C-type natriuretic peptide, adrenomedullin, proadrenomedullin N-terminal peptide, and endothelin-1 (37). Natriuretic peptides play a role in different metabolic processes, including lipid mobilization in human white adipose tissue (38), energy dissipation in brown adipose tissue, the browning of white adipose tissue (39), and fat oxidation in human skeletal muscle (40), possibly influencing whole-body FA metabolism, glucose homeostasis, and insulin sensitivity. We observed a decrease in *Nppb* expression in chow-fed SCD4^−/−^ mice compared with WT mice. This indicates that possible changes in other natriuretic peptides may affect systemic metabolism in SCD4-deficient mice, but this requires further study.

The downregulation of several lipogenic enzymes (eg, acetyl-CoA carboxylase 2, diacylglycerol acyltransferase 1, SCD1, and SCD2) has been shown to improve insulin sensitivity in mice (12, 32, 33, 41). The loss of SCD4 was unable to improve glucose tolerance, which was worsened by HFD feeding, although the HOMA-IR index improved, in which plasma insulin levels were lower in HFD-fed SCD4^−/−^ mice compared with HFD-fed WT mice. This suggests that SCD4 may be directly or indirectly involved in the regulation of insulin gene expression, insulin secretion, and pancreatic islet morphogenesis and β-cell identity, as has been shown for SCD1 (42).

Studies in various mouse models of obesity show that obesity leads to hypertrophic remodeling, fibrosis, and diastolic and systolic dysfunction of the heart (10, 43, 44, 45). The present study showed that the loss of SCD4 reduced EDD and ESD, but did not impair cardiac function or structure under basal conditions. We then showed that 8 weeks of the HFD initiated hypertrophy in WT mice. However, no symptoms of hypertrophy were observed in HFD-fed SCD4^−/−^ mice, suggesting a beneficial effect of SCD4 deficiency and related decreased body adiposity on the myocardium in HFD-treated mice. Moreover, SCD4-deficiency protects cardiomyocytes from 18:0-induced relaxation impairment. The production and secretion of atrial natriuretic factor (encoded by *Nppa*) and brain natriuretic peptide (encoded by *Nppb*) increase in heart failure (46). Additionally, a decrease in α-MHC at the expense of β-MHC is observed in the heart in mice with heart hypertrophy (47). However, changes in cardiac remodeling in HFD-fed WT mice were not associated with changes in expression of the *Nppa*, *Nppb*, *Myh6*, and *Myh7* genes, which were similar to HFD-fed SCD4^−/−^ mice.

However, cardiac remodeling in HFD-fed WT mice may be related to alterations of metabolism in the heart. We found an increase in ATGL activity, indicating an increase in lipolysis in SCD4-deficient cardiomyocytes. ATGL deletion was shown to increase steatosis and fibrosis and decrease the EF in the heart, leading to cardiac dysfunction and premature death in mice (48). The downregulation of ATGL also causes an imbalance of FA uptake and oxidation, promoting the accumulation of ceramides and finally cardiac hypertrophy (49). Furthermore, ATGL deficiency aggravates pressure overload-triggered myocardial hypertrophic remodeling (50). In contrast, cardiac-specific ATGL overexpression prevented TG accumulation in the heart, improved systolic function, and protected against systolic dysfunction and pathological hypertrophy under pressure overload conditions (51). Therefore, an increase in ATGL activity and lipolysis may be partly responsible for the beneficial effects of SCD4 deficiency on the myocardium in HFD-fed mice.

We demonstrated that SCD4 deficiency exerts a protective effect against HFD-stimulated lipid accumulation in the heart. The reduction of cardiac steatosis in SCD4-deficient cardiomyocytes under lipid overload conditions was related to smaller LD size. This suggests that SCD4 downregulation reduces HFD-induced cardiac steatosis by affecting LD dynamics and accelerating LD consumption, likely through the activation of lipolysis. Impairments in cardiac lipolysis have been shown to lead to cardiac steatosis (48, 52, 53), whereas the upregulation of lipolysis reduces cardiac lipid accumulation (52, 54). SCD1 ablation/inhibition decreased cardiac lipid content and increased lipolysis, reflected by a decrease in ATGL inhibitor G0S2 content and a decrease in the ratio of phosphorylated hormone-sensitive lipase at Ser565 to HSL (19). In the present study, we showed that although ABHD5 protein levels increased in the heart in HFD-fed WT mice, the rate of lipolysis was unaltered, likely because of a concomitant increase in G0S2 protein levels. A similar effect was observed in C18:0-treated HL-1 cardiomyocytes. Heier *et al.* (52) showed that an increase in G0S2 expression in the myocardium leads to the inhibition of lipolysis, and the addition of ABHD5 was unable to reverse this effect even partially. Moreover, the phosphorylation of ABHD5 by PKA increases lipolysis through its dissociation from perilipin 5, which inhibits the ATGL-ABHD5 interaction (55). PKA protein levels increased in HFD-fed SCD4-deficient mice, suggesting its involvement in increasing the rate of lipolysis in cardiomyocytes.

Interestingly, we found an increase in ATGL activity in SCD4-deficient hearts and HL-1 cells with the downregulation of SCD4 expression, although ABHD5 and G0S2 protein levels remained unchanged. This suggests a different mechanism of the regulation of ATGL and lipolysis in SCD4-deficient cardiomyocytes. We found an increase in intracellular Ca^2+^ levels in HL-1 cells with the silencing of *Scd4* expression. Some studies showed that Ca^2+^ increases the rate of lipolysis because it is required for the activation of cyclic adenosine monophosphate (cAMP) kinase-dependent lipolysis (28, 29, 30). A possible mechanism depends on the activation of plasma membrane-associated adenylyl cyclases, which produce cAMP, an activator of PKA (56). PKA, in turn, phosphorylates and activates ATGL (57). Thus, we postulated that alterations of calcium metabolism may affect lipolysis in SCD4-deficient cardiomyocytes under basal conditions.

Loss of SCD1 has been shown to inhibit lipogenesis and FA transport (14). These changes resulted in reduced cardiac lipid content in WT, ob/ob, *PPARα* KO mice, and in stearic acid overloaded HL-1 cardiomyocytes with PPARα inhibition (10, 17, 19). We have previously observed that SCD1 and SCD4 activity overlap in the regulation of processes that seek to restore proper functioning of the heart after myocardial infraction (11). Thus, if this overlap also exists under physiological conditions and during HFD feeding, it is possible that the antisteatotic effect of SCD4 deficiency in the heart may also involve changes in lipogenesis, but this requires further study.

A HFD has biphasic actions on mitochondrial biogenesis and function. Initially, it induces the expression of OXPHOS complexes and enhances respiration with a decrease in cardiac efficiency (58, 59), whereas a prolonged HFD leads to impairments in mitochondrial function (32). The overproduction of ROS is partially responsible for the initial activation of mitochondrial respiration (59), but prolonged exposure to high ROS levels leads to mitochondrial damage, a further increase in ROS production, and cardiac dysfunction (31, 32). The 8-week HFD appears to be a transition point because it initiated the development of cardiac hypertrophy in WT mice. Importantly, the HFD did not induce the development of cardiac hypertrophy in SCD4^−/−^ mice. SCD4 deficiency did not affect mitochondrial number or size in the basal state but abolished HFD-induced mitochondrion enlargement. Moreover, the loss of SCD4 attenuated OXPHOS complex I (NADH dehydrogenase) activation and ROS production after the HFD. The inhibition of complex I with metformin partially rescued mitochondrial function, lowered ROS production, and preserved heart function in rats after 12 weeks of a HFD (60). The activation of ROS detoxification by catalase overexpression preserved mitochondrial function, prevented the development of cardiac hypertrophy, and rescued heart function in mice with HFD-induced obesity (31, 61). Thus, the protective effect of SCD4 deficiency on HFD-induced cardiac hypertrophy might be associated with the blunted activation of complex I, which is believed to be one of the main ROS production sites (62).

The other reason for reduced ROS levels in the heart in HFD-fed SCD4^−/−^ mice may be a lower rate of β-oxidation, which affects cellular ROS levels. SCD1 deficiency inhibits FA oxidation (which generates high ROS) and stimulates glucose oxidation (which generates less ROS) (17). More importantly, Gan *et al.* (11) recently showed that the deletion of *Scd4* in the mouse heart reduced β-oxidation that was induced by myocardial infarction. This reduction was associated with decreased activity of ROS producing NADPH oxidase and likely ROS-mediated oxidative stress, which, together with the data presented in this manuscript, indicates that SCD4 is implicated the in the regulation of oxidative stress in cardiomyocytes.

A limitation of our study is that we did not use littermate mice or genotype mixed mice in cages as controls, as this may affect microbiota composition and metabolic phenotype. However, we confirmed our findings in vivo using two in vitro models (HL1 cardiomyocytes with silenced SCD4 expression and primary cardiomyocytes isolated from SCD4^−/−^ mice). In both cell lines, we observed decreased lipid accumulation after 18:0 treatment in cardiomyocytes with reduced *Scd4* expression, which was associated with activated lipolysis. ATGL activity was increased in *Scd4*-silenced HL1 cells and in primary SCD4-deficient cardiomyocytes, as in the heart in SCD4^−/−^ mice. Moreover, in primary culture, we found that 18:0 treatment increased ATGL activity in SCD4-deficient cardiomyocytes, whereas it inhibited ATGL in WT cells. Furthermore, in *Scd4*-silenced HL1 cells, there were no changes in G0S2 protein after 18:0 treatment in contrast to control cells. We observed the same effect of SCD4 deficiency in the heart, where HFD did not affect G0S2 levels in SCD4^−/−^ mice, but increased them in WT mice. This supports our hypothesis that the decreased cardiac steatosis in SCD4^−/−^ mice after HFD is due to the loss of SCD4 in cardiomyocytes. Especially considering that we found no differences in plasma TAG and FFA levels in WT and SCD4^−/−^ mice fed HFD. We have also shown that the preserved contractile function of primary SCD4-deficient cardiomyocytes treated with 18:0 is exclusively due to the loss of SCD4, which also confirms the in vivo results presented here. These in vitro results underline the important role of SCD4 in the regulation of cardiomyocyte metabolism and function.

In conclusion, SCD4 downregulation reduced obesity and had beneficial effects on the heart under HFD conditions, preventing cardiac steatosis, reducing ROS levels, preventing mitochondrial enlargement, and maintaining cardiac function and structure. These effects were partly achieved through an increase in cardiac lipolysis and a decrease in OXPHOS I hyperactivity.

## Data Availability

All data are available in the main text or the supplementary materials.

## Supplemental data

This article contains supplemental data.

## Conflict of interest

The authors declare that they have no conflicts of interest with the contents of this article.
