# Supplementary material for: SCD4 deficiency decreases cardiac steatosis and prevents cardiac remodeling in mice fed a high-fat diet
Source: J Lipid Res. 2024 Jul 31;65(9):100612. doi: 10.1016/j.jlr.2024.100612 (PMC11402454; doi:10.1016/j.jlr.2024.100612)
Supplement: Supplementary Table [file mmc1.pdf]

## Supplementary material

**Table S1.** Primer sequences used for RT-qPCR analysis.

| Gene | Forward primer | Reverse primer |
|---|---|---|
| *Myh6* | GGCACAGAAACACCTGAAGA | CATTGGCATGGACAGCATCATC |
| *Myh7* | ATGTGCCGGACCTTGGAA | CCTCGGGTTAGCTGAGAGATCA |
| *Nppb* | TTTGGGCTGTAACGCACTGA | TTGTGGCAAGTTTGTGCTCC |
| *Nppa* | TGTACAGTGCGGTGTCCAACA | AGAGCCCTCAGTTTGCTTTTC |
| *Scd1* | TACCGCTGGCACATCAACTTC | AACAGGAACTCAGAAGCCCAAAGC |
| *Scd2* | CACTGGGGAGCAGATGTTCG | CAAATACGCGAAGAGACAGGTG |
| *Scd4* | GTTCCAGAGGAGGTACTACAAG | GAGACGCATAAGCTGTGTTG |
| *Ndufv2* | TGGATGGCTACCTATCTCCGCT | GGTACTTCCCAACTGGCTTTCG |
| *Rpl32* | AGTTCCTGGTCCACAATGTCA | GCACACAAGCCATCTACTCATT |

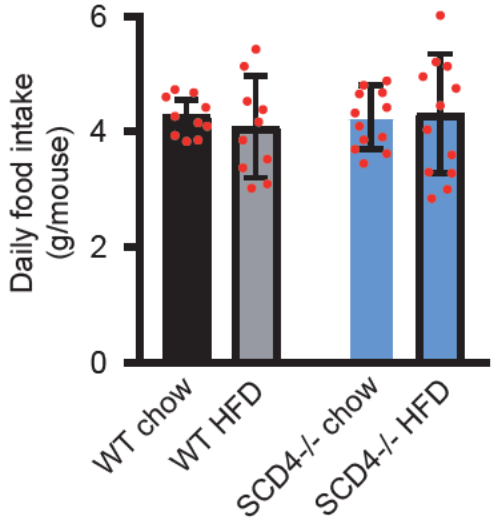


**Fig. S1.** Food intake during the eight weeks of the experiment. The data are expressed as mean ± SD, n = 10-12 mice/group.

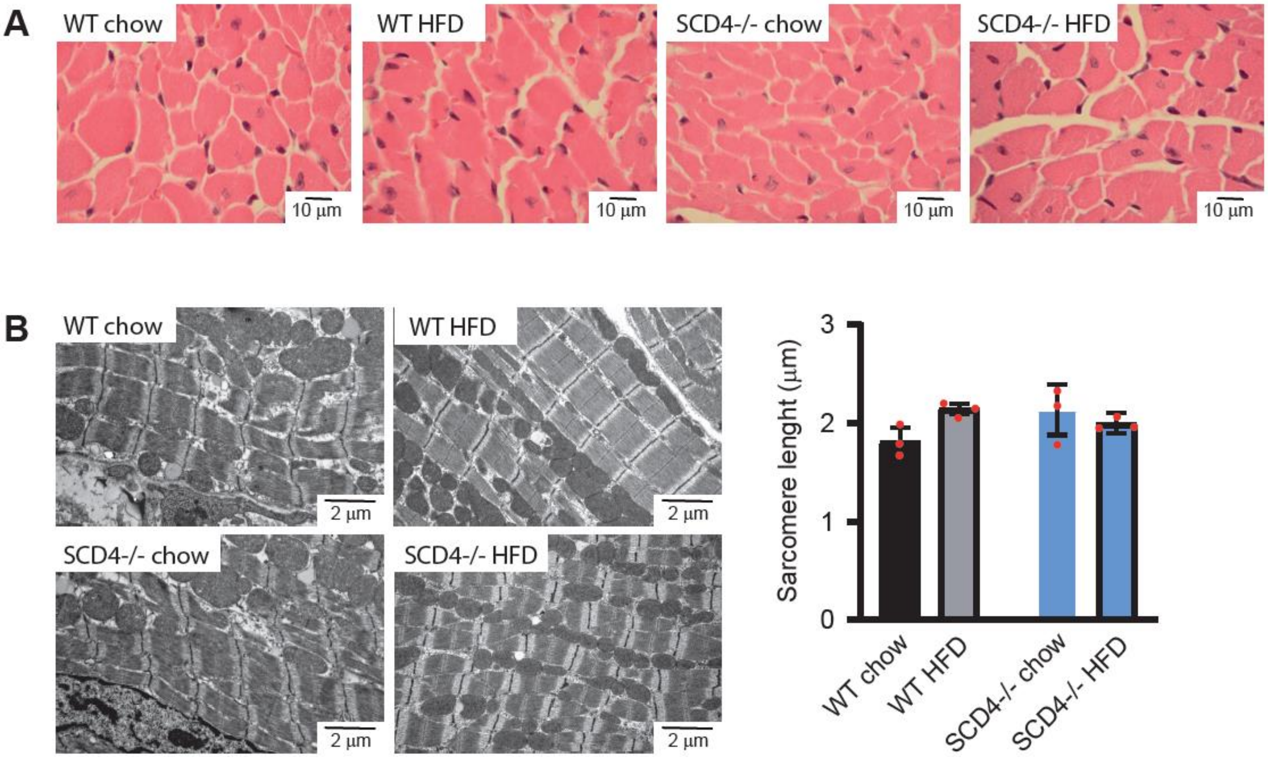


**Fig. S2. (A)** Hematoxylin and eosin staining of mouse left ventricle. Magnification 60 x, scale bar indicates 10 µm. **(B)** Representative electron microscopy images of left ventricular sarcomeres. Magnification 15,000 x, scale bar indicates 2 µm. Sarcomere length ($n > 40$) was measured at 6,000 x magnification electron microscopy images. The data are expressed as mean ± SD, n = 3 mice/group.

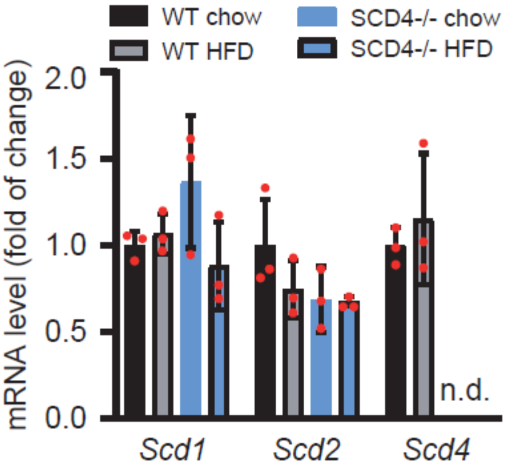


**Fig. S3.** Effect of SCD4 deficiency and HFD on the mRNA levels of individual SCD isoforms expressed in the heart. mRNA level was analyzed using quantitative real–time PCR and $2^{-\Delta\Delta Ct}$ method. The data are expressed as mean ± SD, n = 10–12 mice/group. No significant changes were found.
